# Supplementary material for: Cortical and subcortical signatures of conscious object recognition
Source: Nat Commun. 2021 May 18;12:2930. doi: 10.1038/s41467-021-23266-x (PMC8131711; doi:10.1038/s41467-021-23266-x)
Supplement: Supplementary file 1 — Supplementary Information [file 41467_2021_23266_MOESM1_ESM.pdf]

Cortical and subcortical signatures of conscious object recognition

by Max Levinson, Ella Podvalny, Steven H. Baete, and Biyu J. He

Supplementary Information

Including:

5 supplementary tables and

4 supplementary figures

Recognized > Unrecognized					Unrecognized > Recognized				
Name	# voxels	x	y	z	Name	# voxels	x	y	Z
**ACC**	2002				**L AG**	1207	-46	-60	24
L ACC		-4	8	48	**R AG**	753	48	-62	30
R ACC		4	26	36	**L HC**	196	-26	-22	-20
R amygdala	66	24	-2	-14	**R HC**	114	28	-22	-18
**L aIns**	358	-32	16	10	L IFG	94	-54	22	4
**R aIns**	265	38	18	-2	R IFG	216	58	12	14
**aPCC**	778				R anterior LOC	131	44	-70	4
L aPCC		-4	-32	26	R posterior LOC	58	34	-88	-4
R aPCC		4	-32	26	mid-cingulate	370			
**basal ganglia**	2063				*L mid-cingulate*		-12	-24	38
L NAcc		-10	16	-6	*R mid-cingulate*		8	-24	46
L caudate		-14	12	-12	L MTG	42	-46	-38	-2
L putamen		-22	14	-8	**PCC**	869			
R putamen		18	14	-8	*L PCC*		-10	-54	34
**brainstem**	271				*R PCC*		8	-54	36
L brainstem		-4	-28	-18	**mPFC**	1232			
R brainstem		6	-26	-16	*L mPFC*		-4	60	18
**R IFJ**	160	44	6	30	*R mPFC*		4	52	28
**L IPS**	2629	-30	-58	44	R postcentral	2453	48	-14	54
**R IPS**	1004	34	-66	46	R precuneus	55	12	-56	10
**L MFG**	2049	-44	4	36	subcallosal cortex	149			
**R MFG**	884	42	30	18	*L subcallosal*		-4	24	-16
**R OFC**	338	26	36	-12	*R subcallosal*		4	22	-14
**thalamus**	951				**L SFG**	401			
L thalamus		-16	-18	16	*L anterior SFG*		-16	40	48
R thalamus		12	-4	12	*L posterior SFG*		-20	20	46
**visual cortex**	6267				**R SFG**	113			
L occipital		-10	-92	-6	*R anterior SFG*		18	38	46
R occipital		10	-88	-2	*R posterior SFG*		8	16	58
L ventral temporal		-34	-52	-24	R superior LOC	59	20	-80	34
R ventral temporal		36	-50	-24	L SPL	163	-12	-62	64
					**L STG**	1272	-56	-4	-8
					**R STG**	924	60	-20	-6
					L SMG	101	-62	-34	32
					temporal pole	83	-30	14	-20

Supplementary Table 1. MNI coordinates and sizes of clusters showing significant recognized vs. unrecognized GLM contrast. Clusters are shown in Fig. 3A. Bold names indicate clusters further analyzed in subsequent figures. Sizes are in number of 2mm isotropic voxels, obtained via cluster-based FWE-correction in FSL, with a voxel-defining Z-statistic threshold of $p < 0.001$ and a cluster size threshold of $p < 0.05$. MNI coordinates (mm) either indicate a single voxel with maximum contrast value within the cluster, or one of multiple local maxima (italic names) identified via visual inspection that describe the spatial distribution of the cluster. L: left, R: right, ACC: anterior cingulate cortex, aIns: anterior insula, aPCC: anterior posterior cingulate cortex, NAcc: nucleus accumbens, IFJ: inferior frontal junction, IPS: intraparietal sulcus, MFG: middle frontal gyrus, OFC: orbitofrontal cortex, AG: angular gyrus, HC: hippocampus, IFG: inferior frontal gyrus, LOC: lateral occipital cortex, MTG: middle temporal gyrus, PCC: posterior cingulate cortex, mPFC: medial pre-frontal cortex, SFG: superior frontal gyrus, SPL: superior parietal lobule, STG: superior temporal gyrus, SMG: supramarginal gyrus.

Area	x	y	z
Pons			
L PPN	-8	-26	-18
R PPN	9	-24	-17
L locus coeruleus	-4	-36	-23
R locus coeruleus	4	-36	-23
Midbrain			
Dorsal raphe nucleus	0	-34	-19
Ventral tegmental area	0	-22	-16
Lateral geniculate nucleus (LGN)			
L LGN	24	-25	-6
R LGN	-24	-25	-6

Supplementary Table 2. MNI coordinates (mm) of atlas-derived subcortical structures shown in Fig. 3A. PPN: pedunculopontine nucleus.

Linear mixed model: Recognized category decoding accuracy

Predictors	*Estimates*	*std. Error*	*CI*	*p*
(Intercept)	0.53	0.01	0.50 – 0.55	**<0.001**
Cortical [TRUE]	0.05	0.01	0.03 – 0.08	**<0.001**
Voxel_count	0.01	0.02	-0.03 – 0.05	0.577
Cortical [TRUE] * Voxel_count	0.02	0.02	-0.02 – 0.06	0.314
**Random Effects**				
σ^2	0.01			
$\tau_{00\ \text{Subject}}$	0.00			
$\tau_{11\ \text{Subject.factor(Cortical)TRUE}}$	0.00			
$\tau_{11\ \text{Subject.Voxel\_count}}$	0.00			
$\tau_{11\ \text{Subject.factor(Cortical)TRUE:Voxel\_count}}$	0.00			
ρ_{01}	0.07			
	0.62			
	-0.74			
ICC	0.31			
N_{Subject}	25			
Observations	600			
Marginal R^2 / Conditional R^2	0.111 / 0.382			
log-Likelihood	601.704			

Supplementary Table 3. Effects of ROI location (cortical or subcortical) and voxel count on category decoding accuracy in recognized trials. Recognized object category decoding accuracy was significantly predicted by ROI location (cortical or subcortical), but not ROI voxel count or their interaction, according to a linear mixed model with maximal random effects structure (two-sided t-test on parameter estimates). σ^2: residual (within-subject) variance, τ_{00}: random intercept (between-subject) variance, τ_{11}: random slope (between subject) variance, ρ_{01}: random slope-intercept correlation, ICC: intraclass correlation coefficient. Marginal R^2 includes only fixed effects variance, while Conditional R^2 includes both fixed and random effects variance.

Name	# voxels	x	y	z
real > scr				
L superior frontal gyrus	98	-14	31.1	49.4
L middle temporal gyrus	123	-61	-43	2.35
R superior frontal gyrus	126	9.22	32.6	51
L nucleus accumbens	157	-26	-10	5.42
R angular gyrus	176	53.7	-54	27.1
L anterior insula	205	-35	4.26	-9.3
R inferior frontal gyrus	243	51.2	32.7	0.93
R middle temporal gyrus	307	55.9	-35	-2.6
posterior cingulate	378	-2.2	-50	29.8
mid cingulate	390	-3	-21	39.8
L angular gyrus	677	-53	-47	28.8
R nucleus accumbens	1007	17.5	3.61	-7.7
mPFC	1206	2.71	50.9	13.4
scr > real				
L inferior frontal junction	278	-43	16.1	26.9
dACC	582	-0.9	17.7	42

Supplementary Table 4. MNI coordinates and sizes of clusters showing significant real vs. scrambled GLM contrast. Clusters are shown in Fig. 6A. Sizes are in number of 2mm isotropic voxels, obtained via cluster-based FWE-correction in FSL, with a voxel-defining Z-statistic threshold of $p < 0.01$ and a cluster size threshold of $p < 0.05$. MNI coordinates (mm) indicate the center of gravity of the cluster.

Subject	# runs not acquired	# runs not analyzed	Reason
1	-	1	Error during fMRI acquisition
2	4	-	Subject request to end experiment
6	3	-	Subject request to end experiment
7	-	2	Excessive motion
21	-	1	Excessive motion
24	-	4	Excessive motion

Supplementary Table 5. Reasons for missing task runs in six subjects. Data from 25 subjects were used in analyses reported herein. Except subject #2 and subject #6, all completed the full task including 15 runs. One subject completed 3 extra runs for a total of 18. For subjects #1, #7, #21, and #24, between 1 and 4 task runs were excluded due to acquisition error or excessive motion.

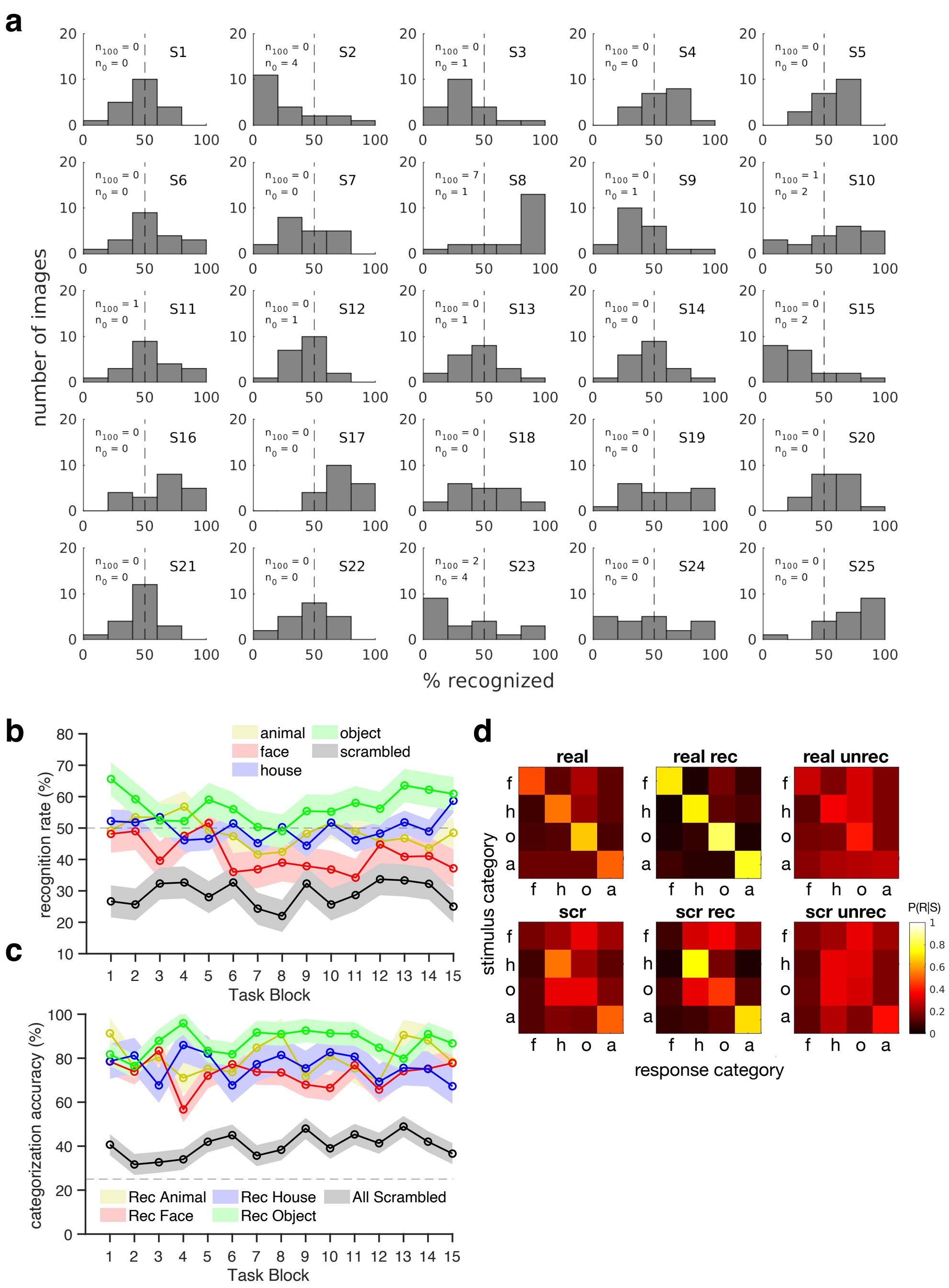

Supplementary Figure 1. Additional behavioral results, averaged across subjects (N=25). A: Histograms of image-specific recognition rates (% of trials reported as recognized) for each subject. Recognition rates were combined into 5 bins of width 20. On average, subjects recognized most images in 46.8 ± 4.7 % of trials (mean ± s.e.m. of the mode, where each subject's mode is the center of their tallest bin), indicating threshold-level perceptual variability. n_0 = number of images never recognized (0%); n_{100} = number of images always recognized (100%). **B:** Percent of trials reported as recognized over 15 blocks of the experiment. Shaded areas indicate standard error of the mean across subjects. **C:** Percent of recognized images that were correctly categorized over 15 blocks of the experiment. Shaded areas indicate standard error of the mean across subjects. **D:** Confusion matrices indicating the probability of subjects to report seeing each category (*response category*) given the actual stimulus category. Data are separated by stimulus type (real object or scrambled) and subjective recognition report. Each four-cell row sums to 1, corresponding to 100% of the trials for a given stimulus category.

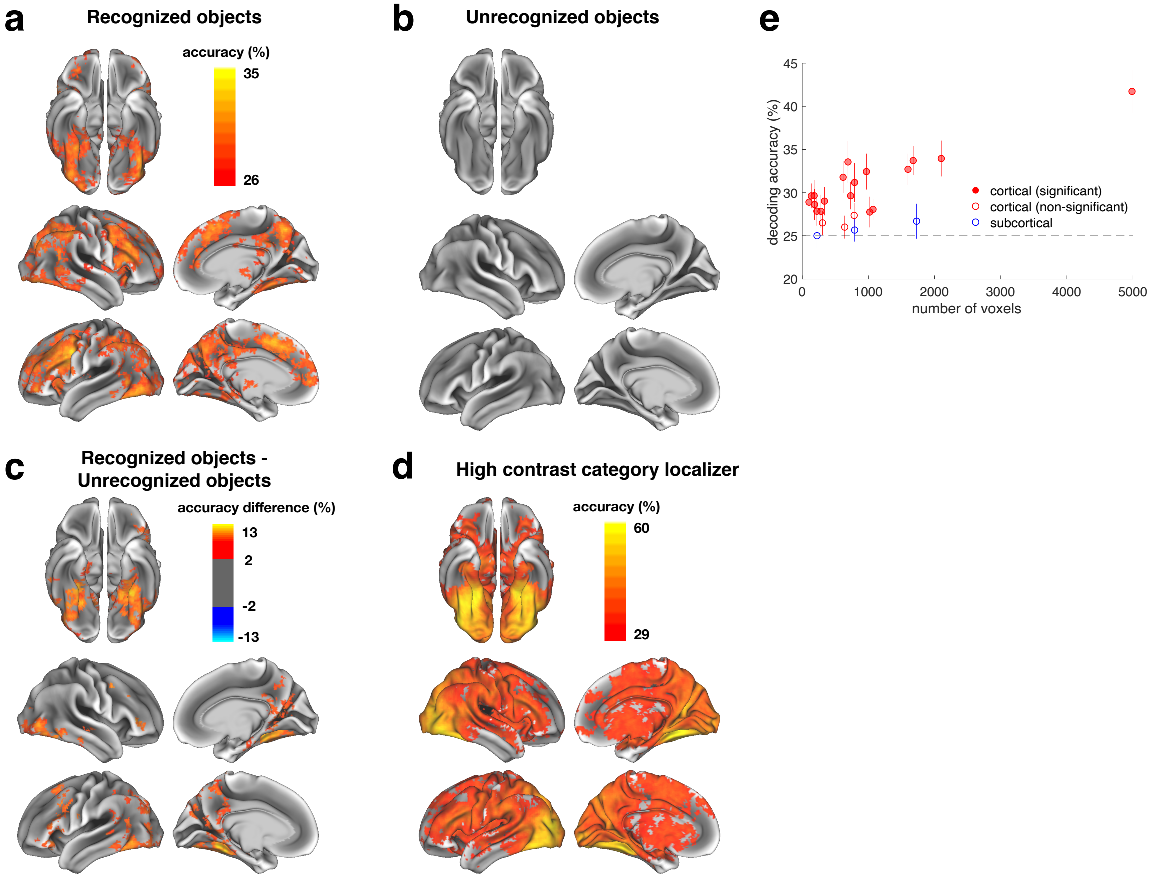

Supplementary Figure 2. Additional decoding analyses. A-D: Whole-brain searchlight analysis of decoding real object category. **A:** Colored areas show voxels for which decoding accuracy for recognized objects alone was significantly greater than chance ($p < 0.05$, cluster-based one-sided permutation test). **B**: No voxels showed significant category decoding for unrecognized objects ($\alpha = 0.05$, cluster-based one-sided permutation test). **C:** Colored areas show voxels for which category decoding performance was significantly higher for recognized versus unrecognized objects ($p < 0.05$, cluster-based one-sided permutation test). **D**: Colored areas show voxels for which category decoding performance was significantly greater than chance in a high-contrast object category localizer run ($p < 0.05$, cluster-based one-sided permutation test). **E:** Relationship between category decoding accuracy and ROI voxel counts, separated into cortical and subcortical regions. Data taken from Figure 4B. Each data point refers to one ROI; error bars denote s.e.m. across subjects ($N = 25$). Filled circles indicate decoding accuracies significantly above chance level as assessed by permutation tests. A linear mixed model identified a significant effect of ROI location (cortical or subcortical) on decoding accuracy ($p < 0.001$), but no significant effect of voxel count or significant interaction between ROI location and voxel count (see Supplementary Table 3).

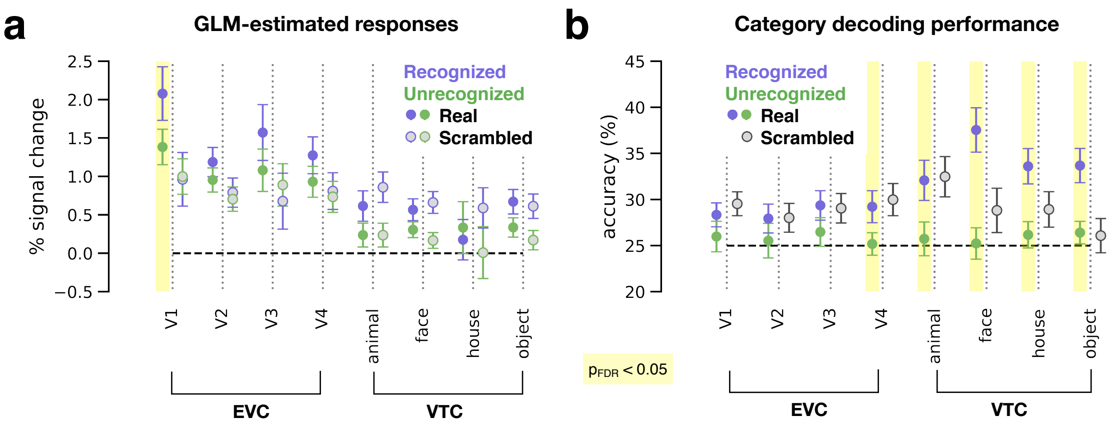

Supplementary Figure 3. Breakdown of data from individually-defined visual cortex ROIs. A: GLM-estimated percent signal change of the stimulus-triggered response, averaged across voxels within V1-V4 (left) and four category-selective ROIs in ventral temporal cortex (right). Shaded columns: significant difference between recognized and unrecognized, two-sided paired t-test, $p < 0.05$, FDR-corrected across all 8 ROIs. **B:** Category decoding accuracy within V1-V4 (left) and four category-selective ROIs in ventral temporal cortex (right). Shaded columns: significant difference between recognized and unrecognized, one-sided label permutation test, $p < 0.05$, FDR-corrected across all 8 ROIs. In all plots, data are averaged across object categories and hemispheres and error bars indicate standard error of the mean across subjects. N: V1 = 23, V2 = 23, V3 = 23, V4 = 22, animal = 18, face = 20, house = 15, object = 18.

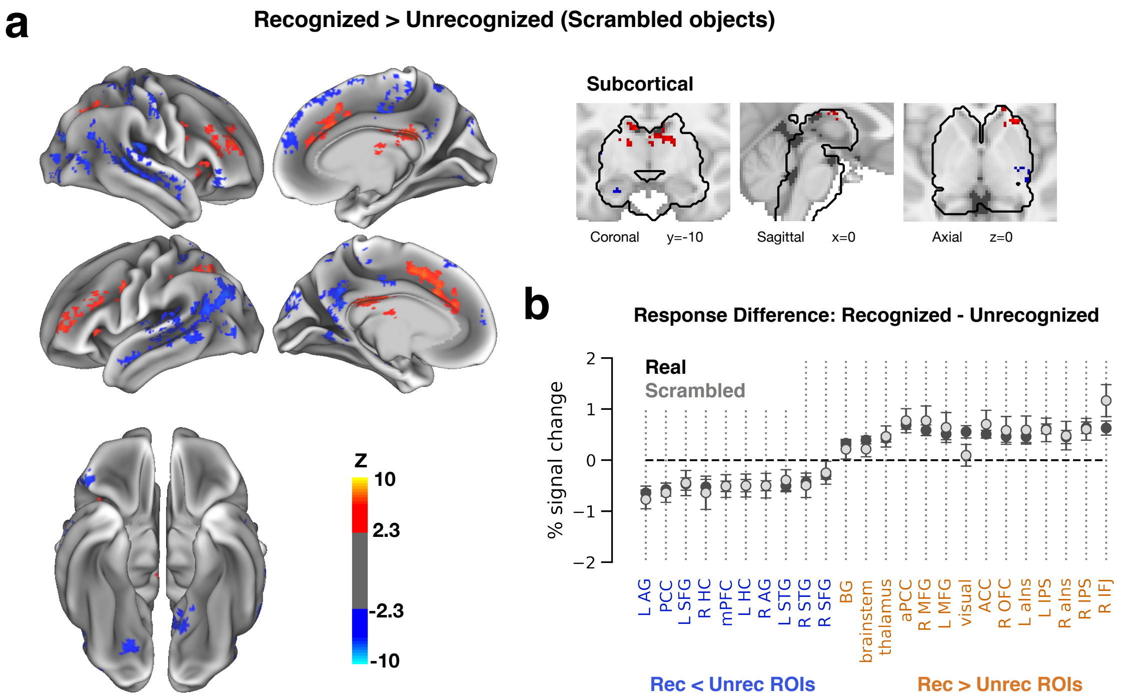

Supplementary Figure 4. General effects of subjectively recognizing scrambled object images. A: Group-level GLM contrast of recognized > unrecognized scrambled object images. Left: data overlaid on a template cortical surface. Right: data presented in subcortical regions only (within black contour), whereas the cortical activity is excluded. Plotted on MNI152 template brain volume. Data were thresholded using cluster-based FWE-correction in FSL, with a voxel-defining Z-statistic threshold of $p < 0.01$ and a cluster size threshold of $p < 0.05$. **B:** Difference in GLM-estimated percent signal change between recognized images and unrecognized images, within ROIs defined by a significant difference for real object images. Black circles show the response difference for real object images, and gray circles indicate that the response difference for scrambled object images is comparable across ROIs to that of real object images. Error bars indicate s.e.m. across subjects (N = 25).
