# Supplementary material for: Cortical and subcortical signatures of conscious object recognition
Source: Nat Commun. 2021 May 18;12:2930. doi: 10.1038/s41467-021-23266-x (PMC8131711; doi:10.1038/s41467-021-23266-x)
Supplement: Supplementary file 3 — Reporting summary [file 41467_2021_23266_MOESM3_ESM.pdf]

nature research

Corresponding author(s): Biyu He

Last updated by author(s): Mar 27, 2021

Reporting Summary

Nature Research wishes to improve the reproducibility of the work that we publish. This form provides structure for consistency and transparency in reporting. For further information on Nature Research policies, see our Editorial Policies and the Editorial Policy Checklist.

Statistics

For all statistical analyses, confirm that the following items are present in the figure legend, table legend, main text, or Methods section.

n/a	Confirmed	
☐	☒	The exact sample size (n) for each experimental group/condition, given as a discrete number and unit of measurement
☐	☒	A statement on whether measurements were taken from distinct samples or whether the same sample was measured repeatedly
☐	☒	The statistical test(s) used AND whether they are one- or two-sided<br>*Only common tests should be described solely by name; describe more complex techniques in the Methods section.*
☐	☒	A description of all covariates tested
☐	☒	A description of any assumptions or corrections, such as tests of normality and adjustment for multiple comparisons
☐	☒	A full description of the statistical parameters including central tendency (e.g. means) or other basic estimates (e.g. regression coefficient) AND variation (e.g. standard deviation) or associated estimates of uncertainty (e.g. confidence intervals)
☐	☒	For null hypothesis testing, the test statistic (e.g. F, t, r) with confidence intervals, effect sizes, degrees of freedom and P value noted<br>*Give P values as exact values whenever suitable.*
☒	☐	For Bayesian analysis, information on the choice of priors and Markov chain Monte Carlo settings
☒	☐	For hierarchical and complex designs, identification of the appropriate level for tests and full reporting of outcomes
☐	☒	Estimates of effect sizes (e.g. Cohen's d, Pearson's r), indicating how they were calculated

Our web collection on statistics for biologists contains articles on many of the points above.

Software and code

Policy information about availability of computer code

Data collection	Behavioral data collection was performed using MATLAB (R2017a). Stimuli were presented using the Psychophysics Toolbox (version 3) in MATLAB.
Data analysis	Behavioral data were analyzed using MATLAB (R2017a). fMRI data were analyzed using FSL (version 5.0.10), AFNI (version 17.2.06), FreeSurfer (version 6.0.0) The Decoding Toolbox (version 3.991) for MATLAB, and the lme4 package (version 1.1) in R (version 4.0.4). Code supporting this study is publicly available at https://github.com/BiyuHeLab/NatCommun_Levinson2021.

For manuscripts utilizing custom algorithms or software that are central to the research but not yet described in published literature, software must be made available to editors and reviewers. We strongly encourage code deposition in a community repository (e.g. GitHub). See the Nature Research guidelines for submitting code & software for further information.

Data

Policy information about availability of data

All manuscripts must include a data availability statement. This statement should provide the following information, where applicable:

- Accession codes, unique identifiers, or web links for publicly available datasets
- A list of figures that have associated raw data
- A description of any restrictions on data availability

The datasets generated and analyzed during the current study are available from the corresponding author by reasonable request. Source data for all main and supplementary figures are provided with this paper.

Field-specific reporting

Please select the one below that is the best fit for your research. If you are not sure, read the appropriate sections before making your selection.

☐ Life sciences ☒ Behavioural & social sciences ☐ Ecological, evolutionary & environmental sciences

For a reference copy of the document with all sections, see nature.com/documents/nr-reporting-summary-flat.pdf

Behavioural & social sciences study design

All studies must disclose on these points even when the disclosure is negative.

Study description	Quantitative experimental study including recordings of visual perceptual behavior and functional magnetic resonance imaging (fMRI) data in healthy adult volunteers.
Research sample	The research sample consisted of healthy human volunteers living in New York City (N=38, 26 females, mean age 27.2, range 20 to 38), including students and non-students. Sample is representative due to an age range covering young adults and both males and females. Rationale for subject selection included availability and sufficiently high level of task performance.
Sampling strategy	A convenience sample was recruited through volunteer subject pools at New York University. We chose a sample size similar to those used in recent published fMRI studies on perception and cognition conducted with healthy human volunteers, such as:<br><br>Carlos González-García, Matthew W. Flounders, Raymond Chang, Alexis T. Baria, and Biyu J. He. Content-specific activity in frontoparietal and default-mode networks during prior-guided visual perception. eLife. 2018 Jul; 7:e36068.
Data collection	The participants were alone inside the fMRI room and experimenters monitored in an adjacent room. Behavioral data were collected via computer (MATLAB, Psychophysics Toolbox). fMRI data were recorded via 7T Siemens MRI scanner and 32-channel head coil.<br>The researchers were not blind to the study hypotheses.
Timing	The data were collected from February 2018 - February 2019.
Data exclusions	1 block from 1 subject was excluded due to a scanner error.<br>2 blocks (1 subject), 1 block (1 subject), and 4 blocks (1 subject) were excluded due to excessive motion artifacts in the fMRI data.<br>Complete data from 3 participants were excluded due to poor performance in the main behavioral task. Exclusion criteria were established prior to the beginning of the study.<br>For decoding analyses that needed training and testing set of trials, we required more than 5 trials in each set.
Non-participation	4 participants did not complete the experiment due to poor performance on a screening task.<br>6 participants declined to complete the experiment due to discomfort.
Randomization	The participants were not allocated into experimental groups.

Reporting for specific materials, systems and methods

We require information from authors about some types of materials, experimental systems and methods used in many studies. Here, indicate whether each material, system or method listed is relevant to your study. If you are not sure if a list item applies to your research, read the appropriate section before selecting a response.

Materials & experimental systems

n/a	Involved in the study	
☒	☐	Antibodies
☒	☐	Eukaryotic cell lines
☒	☐	Palaeontology and archaeology
☒	☐	Animals and other organisms
☐	☒	Human research participants
☒	☐	Clinical data
☒	☐	Dual use research of concern

Methods

n/a	Involved in the study	
☒	☐	ChIP-seq
☒	☐	Flow cytometry
☐	☒	MRI-based neuroimaging

Human research participants

Policy information about studies involving human research participants

Population characteristics	See above
Recruitment	Participants were recruited via flyers, e-mail, and an online research participant recruit platform (researchmatch.org). Since these are common ways of subject recruitment for human neuroscience subjects, we do not expect any resulting potential self-selection bias.
Ethics oversight	The study was approved by the Institutional Review Board of New York University School of Medicine (protocol #15-01323).

Note that full information on the approval of the study protocol must also be provided in the manuscript.

Magnetic resonance imaging

Experimental design

Design type	Task, event-related
Design specifications	15 blocks per subject. Each block includes 24 trials of 8-10 seconds each, with a 6-20 second inter-trial interval (jittered according to an exponential distribution).
Behavioral performance measures	Correct object categorization button press and subjective recognition report button press (yes or no).<br>Task performance was considered acceptable if recognition rate for each category was above 15%, and if categorization accuracy for recognized images was at least 30% higher than for unrecognized images.

Acquisition

Imaging type(s)	Functional and structural MRI
Field strength	7 Tesla
Sequence & imaging parameters	Structural MPRAGE: gradient echo, 3D imaging, FOV 256 mm, matrix size 256x256, slice thickness 1mm, 192 sagittal slices, TE 4.49 ms, TR 3000 ms, flip angle 6 deg.<br>Structural proton density: gradient echo, 3D imaging, FOV 256 mm, matrix size 256x256, slice thickness 1mm, 192 sagittal slices, TR 1760 ms, TE 2.57 ms, flip angle 6 deg.<br>Functional: gradient echo, EPI, FOV 192 mm, matrix size 96x96, slice thickness 2mm (10% distance factor), oblique orientation, TR 2000 ms, TE 25 ms, flip angle 50 deg, multiband factor 2, GRAPPA acceleration 2, phase encoding direction P -> A.
Area of acquisition	Whole brain

Diffusion MRI ☐ Used ☒ Not used

Preprocessing

Preprocessing software	FSL version 5.0.10. Anatomical brain extraction using BET.<br>Functional preprocessing using FEAT. Brain extraction using BET, 3.0 mm FWHM smoothing kernel, high pass filtering with 150 second temporal cutoff, grand mean scaling, slice timing correction.
Normalization	Linear boundary-based registration from functional to anatomical space. Linear registration (12 degrees of freedom) from anatomical to standard MNI152 group space.
Normalization template	MNI152 group standard space
Noise and artifact removal	Artifact removal using independent component analysis (MELODIC): manually inspect 30-40 components that together explain ~75% of variance in the BOLD signal. Artifacts related to motion, arteries, or CSF pulsation were removed.<br>Correction for magnetic field inhomogeneity: the anatomical MPRAGE image was divided by the anatomical proton density image.
Volume censoring	3 parameters of head rotation and 3 parameters of head translation were derived using MCFLIRT in FSL version 5.0.10. Blocks containing excessive motion (>6mm spike in the relative mean displacement timecourse) were removed.

Statistical modeling & inference

Model type and settings	Mass univariate general linear model: fixed effects for each task block, fixed effects for each subject across runs, mixed effects across subjects.

Effect(s) tested

Contrasts: real objects subjectively recognized > baseline, real objects subjectively unrecognized > baseline, scrambled objects subjectively recognized > baseline, scrambled objects subjectively unrecognized > baseline, recognized real objects > unrecognized real objects, real object > scrambled objects.

Specify type of analysis: ☐ Whole brain ☐ ROI-based ☒ Both

Anatomical location(s): Retinotopic and category-selective ROIs were identified using subject-specific visual localizers.

Statistic type for inference (See Eklund et al. 2016)

Cluster-wise: FSL FLAME1. Cluster-defining threshold of $p < 0.01$, cluster size threshold $p < 0.05$.

Correction

FWE cluster correction for whole brain analysis. FDR correction for ROI analysis.

Models & analysis

n/a	Involved in the study
☒	☐ Functional and/or effective connectivity
☒	☐ Graph analysis
☐	☒ Multivariate modeling or predictive analysis

Multivariate modeling and predictive analysis

A linear support vector machine classifier (cost = 1) was trained on voxel-wise beta estimates (from a general linear model) to predict stimulus category from multivoxel patterns. Accuracy was determined using a leave-one-run out cross-validation scheme. Six binary classifications were performed, consisting of all possible pairwise combinations of the four stimulus categories. For each binary classification, the test sample was assigned to one of two categories. Whichever category was chosen in a majority of these binary classifications was selected as the predicted category. To resolve ties, the predicted category was the one that had the maximum decision value summed over all binary classifications. Accuracy was first calculated for each stimulus category separately, and then averaged across categories to produce a final balanced accuracy output per ROI. Significant decoding accuracy at the group-level was assessed using a label permutation test.
